# Migratory Bird-Inspired Adaptive Kalman Filtering for Robust Navigation of Autonomous Agricultural Planters in Unstructured Terrains

**DOI:** 10.3390/biomimetics10080543

**Published:** 2025-08-19

**Authors:** Zijie Zhou, Yitao Huang, Jiyu Sun

**Affiliations:** Key Laboratory of Bionic Engineering, Ministry of Education, Jilin University, Changchun 130022, China; zjzhou23@mails.jlu.edu.cn (Z.Z.); hyt23@mails.jlu.edu.cn (Y.H.)

**Keywords:** bionic algorithm, robust navigation, agricultural planter

## Abstract

This paper presents a bionic extended Kalman filter (EKF) state estimation algorithm for agricultural planters, inspired by the bionic mechanism of migratory birds navigating in complex environments, where migratory birds achieve precise localization behaviors by fusing multi-sensory information (e.g., geomagnetic field, visual landmarks, and somatosensory balance). The algorithm mimics the migratory bird’s ability to integrate multimodal information by fusing laser SLAM, inertial measurement unit (IMU), and GPS data to estimate the position, velocity, and attitude of the planter in real time. Adopting a nonlinear processing approach, the EKF effectively handles nonlinear dynamic characteristics in complex terrain, similar to the adaptive response of a biological nervous system to environmental perturbations. The algorithm demonstrates bio-inspired robustness through the derivation of the nonlinear dynamic teaching model and measurement model and is able to provide high-precision state estimation in complex environments such as mountainous or hilly terrain. Simulation results show that the algorithm significantly improves the navigation accuracy of the planter in unstructured environments. A new method of bio-inspired adaptive state estimation is provided.

## 1. Introduction

**Figure 1:**
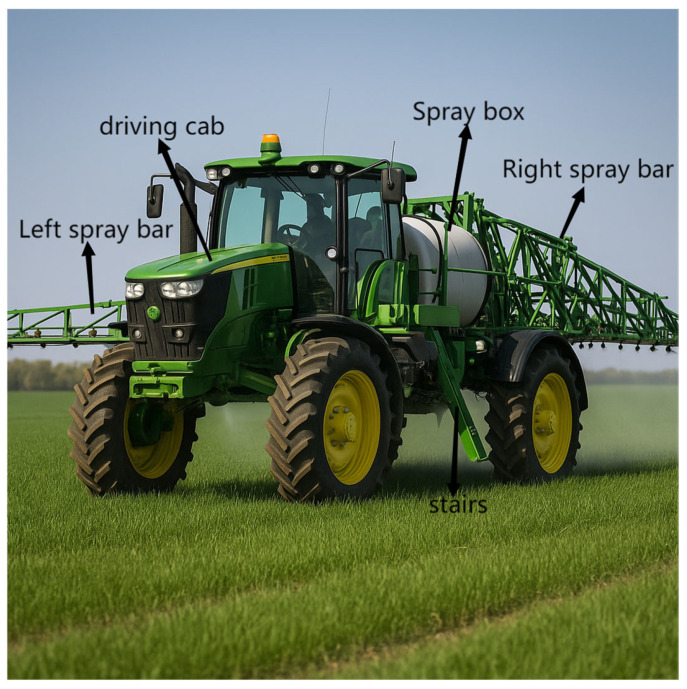
Schematic diagram of an agricultural plant protection spraying machine.

Technological advances in autonomous navigation of agricultural machinery are critical to address labor shortages, increase efficiency, and reduce environmental impact [1,2]. Large self-propelled plant protection machines (Figure 1) play a key role in crop management by reducing waste and environmental pollution through precise application of pesticides and fertilizers [3]. However, precise navigation of these machines in complex agricultural environments faces significant challenges due to changing terrain, obstacles, and dynamic conditions [4,5].

Traditional navigation systems usually rely on Global Navigation Satellite Systems (GNSSs) such as GPS, but signal multipath effects, occlusion, and interference in agricultural environments may affect their performance [6,7]. To overcome these limitations, sensor fusion techniques combine sensors such as GPS, inertial measurement unit (IMU), and laser SLAM to significantly improve navigation accuracy [8,9]. Among them, extended Kalman filtering (EKF) is a powerful tool for integrating multi-source sensor data to estimate machine states with high accuracy.

In recent years, bionics has provided new inspirations in the field of robotics and navigation [10,11], developing innovative robotic systems that mimic natural organisms and enhance performance in various environments. For example, Giovanni Bianch et al. designed snake-inspired aquatic robots that demonstrate better maneuverability in waters [12]. Similarly, Xiaolong He et al. designed quadrupedal robots based on a SLIP model of running animals to achieve efficient and stable mobility on land [13]. In addition, the field of biomimetic optimization has also developed the Migratory Birds Optimization (MBO) algorithm. This algorithm simulates the flight patterns used by migratory birds to solve complex optimization problems. Proposed by Duman et al. in 2012, MBO operates by arranging solutions in a V-shape, where the leader solution explores new neighborhoods, and follower solutions benefit from shared improvements, reducing computational redundancy [14]. Key advantages of MBO include its efficiency in balancing exploration and exploitation, lower sensitivity to initial parameters compared to traditional metaheuristics, and superior performance in discrete optimization tasks due to its neighborhood-sharing mechanism, which emulates birds’ energy conservation during migration [14,15]. Recent achievements highlight MBO’s versatility. For instance, in 2024, an enhanced MBO hybridized with simulated annealing achieved state-of-the-art results in global optimization benchmarks, outperforming standard genetic algorithms by up to 15% in convergence speed [15]; another 2024 study applied an improved MBO to closed-loop supply chain network design, reducing costs by 12% over particle swarm optimization (PSO) in real-world logistics scenarios [16]. Compared to other algorithms like PSO or ant colony optimization (ACO), MBO excels in problems with high-dimensional search spaces, as it requires fewer iterations (e.g., 20–30% less than PSO in quadratic assignment problems) and avoids premature convergence through dynamic benefit-sharing [14,17]. In the context of navigation and filtering, MBO has been explored for parameter tuning in Kalman filters, such as optimizing noise covariances in UAV systems inspired by bird migration senses [18]. This is consistent with the biomimetic EKF proposed in this article.

Migratory birds exhibit remarkable navigational abilities during migration, being able to travel across thousands of kilometers to reach their destinations with precision. This ability stems from their integration of multimodal sensory information from visual landmarks (e.g., mountains, rivers), geomagnetic fields, and somatosensory balance (e.g., acceleration and angular velocity perceived by the vestibular system) [19,20]. For example, under clear weather, migratory birds mainly rely on visual landmarks for localization; however, in cloudy or stormy weather, they dynamically adjust their strategies by increasing their reliance on geomagnetic fields or somatosensory sensations to adapt to environmental perturbations [21]. In this study, based on the above neuronavigation characteristics of migratory birds, a bionic extended Kalman filter (EKF) algorithm is proposed, with the following mapping relationship.

Sensory confidence is mapped from the frequency of discharge or the intensity of response of migratory birds’ sensory neurons to external stimuli. For example, when the visual information is clear, the activity of the relevant neurons of migratory birds is enhanced, corresponding to a higher confidence level; however, in low visibility environments, such as windy and rainy conditions, the neural discharge of this channel is weakened, corresponding to a decrease in the confidence level [22,23].

The sensitivity coefficients correspond to the slopes or gains of the different sensory systems of migratory birds in response to signal changes, i.e., the degree of sensitivity of neurons to input stimuli. For example, the vestibular system of migratory birds responds faster than the geomagnetic system during vigorous maneuvers and has a higher sensitivity coefficient [24,25]. Thus, the sensitivity coefficients reflect the response properties inherent in the physiological structure of the sensory channels in migratory birds [26].

Dynamic process noise regulation coefficients model the adjustment of migratory birds’ tolerance to predictive mechanisms when external signals become weak or ambiguous, corresponding to the regulation of uncertainty in the feedforward and feedback pathways in the migratory bird’s nervous system with respect to intrinsic state prediction mechanisms [27]. Such regulation is reflected in the enhanced tolerance and weighting of the neural system’s own motion model when it does not perceive the outside adequately [28].

Time-varying measurement noise tuning, on the other hand, maps from the ability to learn and adapt to short-term learning of sensory channels in the nervous system. Examples include neuromodulatory behavior under repeated stimuli in migratory birds [27].

Although a variety of organisms possess navigation capabilities (e.g., insect polarized light navigation, ant path integration) [29,30], migratory bird-specific multimodal perception and large-scale terrain environment adaptation are more useful in unmanned agricultural systems [22]. Compared to insect-based bionic navigation, the following observations are noted:

- Insect-dependent polarized light modes are unstable in agricultural overcast and hazy environments [30];
- Migratory birds fuse vision, geomagnetism, and somatosensory senses to possess stronger environmental generalization [23,26];
- Bird neural systems are robust and modellable in navigation switching strategies, which are easy to map to filter parameter adjustment [31].

The navigation of autonomous agricultural seeders is a critical task that requires precise and robust estimation of their own state to ensure efficient and accurate operation in dynamic and often unpredictable environments. The recent advancements in Kalman filtering technology demonstrates its ability to handle nonlinear and partially known dynamic systems. For instance, Revach et al. [32] introduced KalmanNet, a method that combines neural networks with Kalman filtering to effectively handle non-linear dynamics when only partial information is available. Similarly, Harl et al. [33] developed a neural network-based modified state observer to estimate uncertainties in orbit determination, demonstrating the potential of neural networks to enhance state estimation in the presence of model uncertainties. Furthermore, Zhou et al. [34] proposed an adaptive order-switching Kalman filter that leverages deep neural networks for nonlinearity detection, offering a mechanism to balance computational efficiency and estimation accuracy by dynamically adjusting the filter’s complexity. In this paper, we propose a bionic extended Kalman filter algorithm for a planting machine that can be used by the planting machine to estimate its own state in real time under the complex environment. The main innovations of this paper are as follows:

- A mathematical model of the neural navigation mechanism of migratory birds, including a proposed sensory weight mapping formula with structural fusion of state residuals;
- A time-varying fusion and dynamic noise adjustment mechanism, combined with soft switching idea to improve robustness;
- Fusion of deep learning classifiers to realize online environment recognition and parameter self-tuning, breaking through the limitations of traditional EKF that requires manual parameter tuning.

The rest of this paper is organized as follows: Section 2 models the system of plant protection machine, Section 3 introduces the bionic EFK algorithm, Section 4 verifies the performance of the algorithm through comparative experiments, and Section 5 concludes the study and proposes future application scenarios.

## 2. System Modeling

### 2.1. Definition of State Vector

In order to accurately describe the motion characteristics of the agricultural plant protection machine in complex terrain, the state vector is designed as a nine-dimensional vector containing position, velocity, and attitude. The state vector x is defined as follows:

(1) $$x\;=\;\left[\begin{array}{c} p_{x}\\ p_{y}\\ p_{z}\\ v_{x}\\ v_{y}\\ v_{z}\\ \phi \\ \theta \\ \psi \end{array}\right]$$

where $p_{x},\;{\;p}_{y},{\;p}_{z}$ (unit: meter) denotes the position coordinates of the planting machine in 3D space; $v_{x},{\;v}_{y},{\;v}_{z}$ (unit: m/s) denotes the velocity components along the three axes; and ϕ,  θ,  ψ (unit: radian) denotes the roll angle, pitch angle, and yaw angle, respectively, which are used to describe the spatial orientation of the planting machine. This state vector design can comprehensively capture the motion state of the planting machine, laying the foundation for subsequent dynamic modeling and state estimation.

### 2.2. Nonlinear Dynamic Model

The motion of agricultural plant protection aircraft in complex terrain (such as hills or mountains) exhibits significant nonlinear characteristics, so a nonlinear dynamic model is used to describe the change of its state with time. The dynamic model is defined as follows:

(2) $$\dot{x}\;=\;f^{\prime }(x,u)$$

where u is the control input vector containing the three-axis acceleration $a_{x},a_{y},a_{z}$ in meters per second^2^ and the angular velocity p, q, r in radians per second, which are supplied by the inertial measurement unit IMU. The nonlinear function $f^{\prime }(x,u)$ is specifically defined as follows:

(3) $$f^{\prime }(x,u)\;=\;\left[\begin{array}{c} v_{x}\\ v_{y}\\ v_{z}\\ a_{x}\\ a_{y}\\ a_{z}\\ p\;+\;qsin\phi tan\theta \;+\;rcos\phi tan\theta \\ qcos\phi \;-\;rsin\phi \\ qsin\phi /cos\theta \;+\;rcos\phi /cos\theta \end{array}\right]$$

The function describes the change rule of the state vector, and the position component is updated with velocity ${\dot{p}}_{x}\;=\;v_{x},{\;\dot{p}}_{y}=v_{y},\;\;{\dot{p}}_{z}\;=\;v_{z}$ to indicate the cumulative change of position with velocity. The velocity component is updated with acceleration ${\dot{v}}_{x}\;=\;a_{x},\;\;{\dot{v}}_{y}\;=\;a_{y},{\dot{\;v}}_{z}\;=\;a_{z}$ to indicate the effect of acceleration on velocity, and the attitude update equation is based on Euler’s angular kinematics, which calculates the rate of change of the attitude angle through the angular velocity p,  q,  r. This nonlinear model can accurately describe the dynamic behavior of the plant protection aircraft in complex environments.

### 2.3. Measurement Model

The measurement model is used to associate sensor observations with state vectors by integrating data from GPS and laser SLAM to provide accurate observation information. Measurement vector $z_{k}$ is defined as follows:

(4) $$z_{k}\;=\;h(x_{k})\;+\;v_{k}$$

where the measurement vector $z_{k}$ contains 3D position measurements provided by GPS, position and attitude measurements provided by SLAM, and velocity measurements provided by IMU (obtained by integration); $h(x_{k})$ is a nonlinear measurement function representing the theoretical mapping relationship between the state vector x*_k_* and the measurement vector $z_{k}$; and $v_{k}$ is the measurement noise obeying a zero-mean Gaussian distribution with a covariance matrix R. This model provides a reliable observation basis for state estimation by fusing multi-source sensor data.

## 3. Biomimetic Extended Kalman Filter (EKF) Algorithm

### 3.1. Standard Extended Kalman Filter

The standard extended Kalman filter (EKF) is a widely used method for state estimation in nonlinear systems, providing a recursive solution by linearizing the system dynamics and measurement models around the current estimate. The algorithm consists of two main steps: prediction and update.

#### 3.1.1. Prediction Step

The prediction step of the extended Kalman filter (EKF) predicts the next moment state of the plantation machine through a nonlinear dynamic model and evaluates its uncertainty through the covariance matrix. The state prediction equation is expressed as follows:

(5) $${\hat{x}}_{k}^{-}\;=\;f({\hat{x}}_{k\;-\;1},u_{k})$$

where ${\hat{x}}_{k}^{-}$ is the a priori state estimate, ${\hat{x}}_{k\;-\;1}$ is the a posteriori state estimate from the previous moment, and $u_{k}$ is the current control input, including the acceleration and angular velocity measured by the IMU. The function is calculated by the fourth-order Runge-Kutta method.

The covariance prediction equation is expressed as follows:

(6) $$P_{k}^{-}\;=\;F_{k}P_{k\;-\;1}F_{k}^{T}\;+\;Q_{k}$$

where $P_{k\;-\;1}$ is the covariance matrix of the previous moment, which describes the uncertainty of the state estimation; $Q_{k}$ is the process noise covariance matrix, which reflects the effect of system noise; and $F_{k}\;=\;\frac{\partial f}{\partial x}{|}_{{\hat{x}}_{k\;-\;1}}$ is the state transfer Jacobian matrix. It is defined as follows:

(7) $$F_{k}=\left[\begin{array}{ccc} 0_{3\;\times \;3} & I_{3\;\times \;3} & 0_{3\;\times \;3}\\ 0_{3\;\times \;3} & 0_{3\;\times \;3} & 0_{3\;\times \;3}\\ 0_{3\;\times \;3} & 0_{3\;\times \;3} & \frac{\partial \dot{\Phi }}{\partial \Phi } \end{array}\right]$$

where, $\Phi \;=\;{\left[\phi ,\theta ,\psi \right]}^{T}$. The Jacobi matrix for the pose part is expressed as follows:

(8) $$\partial \dot{\Phi }/\partial \Phi \;=\;\left[\begin{array}{ccc} qcos\phi tan\theta \;-\;rsin\phi tan\theta & (qsin\phi \;+\;rcos\phi ){sec}^{2}\theta & 0\\ -qsin\phi \;-\;rcos\phi & 0 & 0\\ qcos\phi /cos\theta \;-\;rsin\phi /cos\theta & (qsin\phi \;+\;rcos\phi )tan\theta /cos\theta & 0 \end{array}\right]$$

This matrix reflects the dynamic coupling between the state variables by calculating the partial derivatives of the nonlinear function $f^{\prime }(x,u)$, ensuring that the prediction step accurately captures the nonlinear properties.

#### 3.1.2. Update Steps

The update steps for the EKF use sensor observations to correct the predicted state and optimize the state estimation accuracy. The state update equation is expressed as follows:

(9) $${\hat{x}}_{k}\;=\;{\hat{x}}_{k}^{-}\;+\;K_{k}(z_{k}\;-\;h({\hat{x}}_{k}^{-}))$$

where ${\hat{x}}_{k}$ is the a posteriori state estimate, and $z_{k}\;-\;h({\hat{x}}_{k}^{-})$ is the measurement residual, which represents the deviation of the observed value from the predicted value. The measurement prediction is calculated by $h({\hat{x}}_{k}^{-})$, which generates the expected observation based on the a priori state. $K_{k}$ is the Kalman gain. The Kalman gain is calculated as follows:

(10) $$K_{k}\;=\;P_{k}^{-}H_{k}^{T}{\left(H_{k}P_{k}^{-}H_{k}^{T}\;+\;R\right)}^{-1}$$

where $H_{k}\;=\;\frac{\partial h}{\partial x}{|}_{{\hat{x}}_{k}^{-}}$ is the Jacobi matrix of the measurement function. It is defined as follows:

(11) $$H_{k}\;=\;\left[\begin{array}{ccc} I_{3\;\times \;3} & O_{3\;\times \;3} & O_{3\times 3}\\ I_{3\;\times \;3} & O_{3\;\times \;3} & O_{3\;\times \;3}\\ O_{3\;\times \;3} & O_{3\;\times \;3} & I_{3\;\times \;3} \end{array}\right]$$

This matrix maps the state vectors to the observation space, reflecting the direct effect of IUM, GPS, and SLAM measurements on position and attitude; *R* is the measurement noise covariance matrix, which describes the sensor noise characteristics. The covariance update equation is expressed as follows:

(12) $$P_{k}\;=\;(I\;-\;K_{k}H_{k})P_{k}^{-}$$

where I is the unit matrix, and the updated covariance matrix $P_{k}$ reduces the uncertainty. This step optimizes the state estimation by fusing the observations and adapts to the complexity of the nonlinear system.

### 3.2. Bio-Inspired Adaptive Mechanisms

Building upon the standard EKF described in Section 3.1, this subsection introduces the novel bio-inspired enhancements inspired by the multimodal navigation strategies of migratory birds. These adaptations improve the robustness and accuracy of state estimation in complex agricultural environments by dynamically adjusting sensor fusion weights, process noise covariance, and measurement noise covariance.

#### 3.2.1. Sensor Fusion Weights Adjustment Model

To optimize the fusion effect of multi-source sensor data, this paper proposes a dynamic weight adjustment model based on the neural mechanism of migratory birds, which abstracts a set of engineering-implementable sensory selection mechanisms from the multimodal navigation behavior of migratory birds. Biological studies have shown that migratory birds assign different navigation weights to different sensory channels (visual, geomagnetic, and somatosensory) according to the environmental conditions during migration, and their strategy switching and neural activities are reflected in the dynamic changes of neural firing patterns in multisensory integration areas (e.g., the vestibular system, the optic cortex, and the parietal cortex) at the physiological level. Although this regulation has not been uniformly modeled as a specific neurodynamic system, its behavioral response patterns have been confirmed to be predictable by neuroecological experiments. In this paper, we adopt an abstract modeling approach at the level of behavioral mechanisms to transform the migratory birds’ prioritization of sensory channels in different environments into a set of soft selection weighting formulas. Let the three sensory channels be G_vision_ (visual landmarks), G_mag_ (geomagnetic navigation), and G_vest_ (somatosensory balance), with sensory weights *w*_1_, *w*_2,_ and *w*_3_, respectively, and their weights are assigned at any time t. The weights are then calculated as follows:

(13) $$w_{i}(t)\;=\;\frac{\exp(\alpha_{i}\cdot s_{i}(t))}{\sum_{j\;=\;1}^{3}\exp(\alpha_{j}\cdot s_{j}(t))}$$

where $s_{i}(t)$ is the confidence signal strength of the ith sensory channel and $\alpha_{i}$ is the sensitivity coefficient of the corresponding channel. This mechanism is equivalent to a softmax neural strategy, which is able to dynamically perceive the environmental advantages and disadvantages and switch the dominant sensory channel.

**Figure 2:**
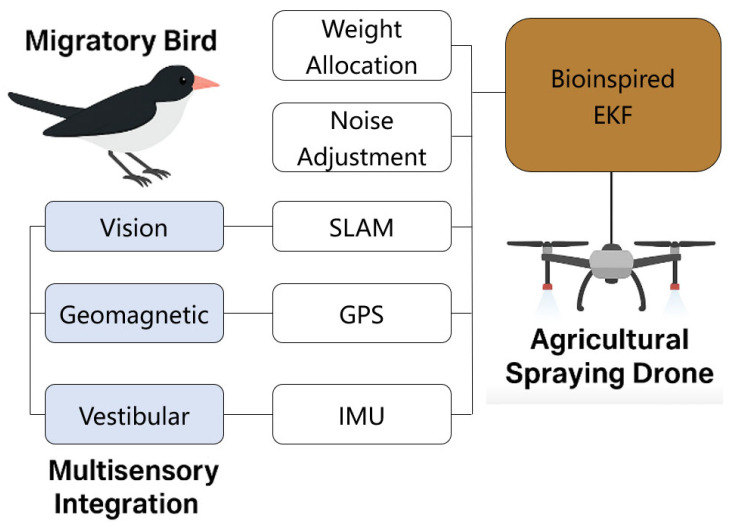
Schematic diagram of the concept of multisensory integration.

In the engineering implementation, $s_{i}(t)$ can be estimated from the historical residual variance, and $\alpha_{i}$ can be learned from an online learning module. This modeling mechanism implements a formal mapping from the migratory bird neural strategy to adaptive filter weight assignment. The conceptual schematic is shown in Figure 2.

#### 3.2.2. Dynamic Adjustment Model for Noise Covariance

Based on the behavior of migratory birds in adjusting their navigation strategies under different weather conditions, a mechanism for dynamically adjusting the process noise covariance is proposed. The dynamic adjustment formula is expressed as follows:

(14) $$Q_{k}\;=\;Q_{0}\;+\;\alpha \cdot \mathrm{var}(u_{k})$$

where $Q_{0}$ is the underlying noise covariance matrix; $\mathrm{var}(u_{k})$ represents the inherent noise of the system and the variance of the control inputs (e.g., acceleration and angular velocity), reflecting the strength of the environmental perturbation; and α is the adjustment coefficient controlling the magnitude of the adjustment.

In the neural mechanism of migratory birds, if the visual or geomagnetic trust decreases, the system will rely more on the feedback from the somatosensory system. The somatosensory feedback is proprioceptive in nature and has a greater impact on the prediction component. Therefore, migratory birds will increase the tolerance for process uncertainty when sensory trust is unclear. When the overall reliability of the external sensory observations decreases (e.g., a severe failure of the GPS), it should be boosted by α to enhance the process noise covariance to avoid overconfidence in the prediction results. The acquisition of the conditioning factor α depends on the assignment of migratory bird sensory weights, thus creating an overall confidence indicator:

(15) $$w_{\mathrm{sensor}}\;=\;\sum_{i\;=\;1}^{n}\gamma_{i}\cdot w_{i}$$

where $w_{i}$ is the *i*-th sensory weight, $\gamma_{i}$ is the base confidence factor for each sense (e.g., 0.6 for SLAM, 0.3 for GPS, and 0.1 for IMU), and $w_{\mathrm{sensor}}$ represents the overall confidence in the current system’s observations of the senses. Then, α is defined as follows:

(16) $$\alpha \;=\;\alpha_{\max}\cdot (1\;-\;w_{\mathrm{sensor}})$$

This formulation is based on behavioral abstraction modeling of migratory bird sensory trust switching behavior and reflects an engineering-controlled linear inverse relationship. When overall sensory trust $w_{\mathrm{sensor}}$ is higher (i.e., the sensory channel on which the system is currently relying is more trustworthy), α should be smaller, the process noise covariance is lower, and the prediction process is more “confident”. In contrast, when sensory trust credibility is low, α rises, and the filter becomes more resilient to potential state drifts, increasing robustness.

The design of the dynamic tuning mechanism maps the bionic principle of migratory bird navigation. The differential setting of the maximum tuning coefficient $\alpha_{max}$ simulates the migratory bird’s trust in senses under different environments. A low value of $\alpha_{max}$ represents trust in senses under stable environments, while a high value of α corresponds to distrust in senses under harsh conditions. This mapping from biological behavior to algorithmic parameters is essentially to transform the robustness mechanism formed by natural evolution into adaptive rules of engineering systems. In practical engineering, the value of the adjustment coefficient $\alpha_{max}$ should be determined in combination with the dynamic characteristics of the system task and the complexity of the environment. For agricultural scenarios that prioritize accuracy (e.g., open field operation), the value range is generally 0.05–0.20 to ensure the stability of state propagation. For applications with higher robustness requirements (e.g., hilly woodland, post-disaster search, etc.), the value can be appropriately increased to 0.20–0.50 to enhance tolerance to sensor failure and environmental perturbation. In highly dynamic systems, the value of the adjustment factor should be determined in conjunction with the dynamic characteristics of the system task and the complexity of the environment. In highly dynamic systems (e.g., UAVs or fast maneuvering robots), where the process changes drastically, it is usually necessary to set $\alpha_{max}$ between 0.30 and 0.80 to ensure that the filter does not become overconfident in fast response. This is shown in Table 1.

#### 3.2.3. Time-Varying Measurement Noise Estimation

A time-varying measurement noise covariance estimation method is proposed to dynamically assess the credibility of each sensory information based on migratory birds’ real-time perception of environmental feedback (e.g., changes in wind speed and airflow) through the nervous system. The fused residuals are formulated as follows:

(17) $$r_{k}\;=\;\sum_{i\;=\;1}^{n}w_{i}\cdot \left(z_{k}^{\left(i\right)}\;-\;h^{\left(i\right)}({\hat{x}}_{k}^{-})\right)$$

where *i* represents different sensors.

The measurement noise covariance estimation equation is expressed as follows:

(18) $$R_{k}\;=\;(1\;-\;\beta )R_{k\;-\;1}\;+\;\beta \mathrm{Cov}[r_{k}]$$

where $R_{k\;-\;1}$ is the covariance of the measurement noise at the previous moment, and β is a smoothing factor that controls the update rate. The selection of β requires a balance between stability and adaptability. Usually, in environments with relatively stable noise characteristics (such as plain terrain under clear weather), the value of β is small, ranging from 0.01 to 0.1, to prevent the filter from overreacting to temporary noise fluctuations or outliers, thereby maintaining the stability of the estimation. However, in scenarios with rapidly changing noise characteristics (such as windy and rainy weather in hilly terrain), a larger β value is required to accelerate the adjustment speed of $R_{k}$, so that the filter can quickly adapt to new noise conditions and maintain the accuracy of state estimation. In practical applications, the selection of β can be determined through empirical testing or real-time performance monitoring. For example, if the estimation error of the filter increases over time, it may indicate that the current value of β is not sufficient for $R_{k}$ to quickly adapt to noise changes. In this case, β can be appropriately increased. On the contrary, if the filter exhibits instability or is overly sensitive to noise, β should be reduced to enhance stability. The method for determining the smoothing factor is based on sliding window statistics. Using the statistical characteristics of the residuals from the last 10 measurements, if the mean of the residuals is small (representing an environment with slowly changing noise), the value of β is reduced to reduce the sensitivity of the filter to short-term disturbances. If the statistical residual mean is large (representing an environment with rapidly changing noise), the value of β is increased to ensure that the update speed is slowed down when the noise fluctuates greatly, making the filter more adaptable. The adjustment of the β value helps the filter maintain a low average position error and improves the robustness of the algorithm.

## 4. Experimental and Simulation Analysis

In order to comprehensively verify the performance of the proposed bionic EKF algorithm, this section compares and analyzes its performance with traditional algorithms in complex agricultural environments using multi-scenario simulation experiments and introduces the UKF, the PF, and a bionic navigation algorithm based on the path integral as comparisons. UKF uses the traceless transformation to replace the Jacobi computation, which improves the nonlinear adaptability. PF uses particle sampling to characterize the state distribution, which is robust but with high computational complexity. The bionic path integral algorithm imitates the insect to estimate the relative displacement, which is suitable for short-distance navigation. In this paper, the algorithms achieve self-adaptation to the complex environment by dynamically adjusting the process noise covariance and time-varying measurement noise covariance and optimizing the sensor fusion weights. The evaluation index is centered on the mean position error (MPE), supplemented by algorithm robustness analysis. In order to realize the ability to automatically adapt to the environment, an LSTM-based environment classification module is introduced. The input is the sensor residual time series, and the output is the current scene category, which is further mapped to the adjustment of α and β parameters. The structure of the classifier is described as follows: input layer (time window = 10 steps) → two-layer LSTM (hidden element = 32) → fully connected mapping → softmax output. Predictive labeling controls weighting strategy switching to reach fully online adjustment.

The effectiveness of the bionic mechanism is verified by comparing the performance of the algorithm in different scenarios. The experimental design covers different combinations of terrain and weather to simulate the variable conditions in real agricultural operations. The simulation platform is implemented based on Python 3.9, and each combination of environments is run for 500 steps to ensure the statistical significance of the results.

### 4.1. Experimental Design

The experimental environment is divided into two types of terrain, plains and hills, and combined with three types of weather conditions, sunny, heavy rain, and windy and rainy, to form a total of six test scenarios. The experimental data utilized in this study are generated through simulations in Python 3.9, employing synthetic datasets that model real-world sensor readings from GPS, IMU, and laser SLAM under varying environmental conditions. Specifically, the data consist of time-series sequences (500 steps per run, with a step duration of 0.1 s simulating 50 s of operation) including position (x, y, z in meters), velocity (m/s), attitude (radians), and control inputs (acceleration in m/s^2^ and angular velocity in rad/s). These datasets are synthesized using Gaussian noise models augmented with multiplicative factors to replicate physical disturbances. Baseline noise is drawn from zero-mean distributions with covariances calibrated from real agricultural sensor benchmarks (derived from field tests) [3] and then amplified as per Table 2 to account for terrain (e.g., hills introducing occlusion via ray-tracing simulation) and weather (e.g., rain adding multipath via random perturbations up to ±30%). Random perturbations are generated using NumPy’s random normal function to introduce variability, ensuring statistical relevance to unstructured farmland scenarios like those in hilly Chinese orchards or Midwestern US plains [5]. The noise amplification factor of the sensor is shown in Table 2.

### 4.2. Simulation Results and Analysis

**Figure 3:**
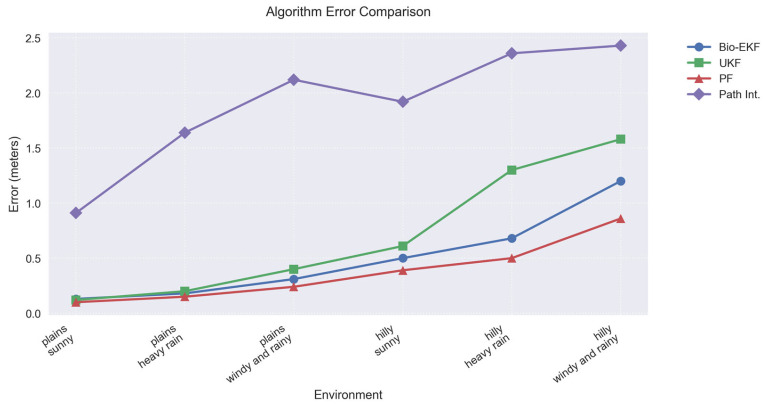
Comparison of average position error.

The experimental results, including mean position errors (MPE), time consumption, and error variance, are presented in Figure 3 (comparison of average position error across scenarios), Table 3 (single-step time consumption in milliseconds), and Table 4 (MSE and 95% confidence intervals), demonstrating the algorithm’s superior accuracy and efficiency in simulated complex agricultural environments. These metrics are derived from averaged outcomes over 100 Monte Carlo runs per scenario to ensure statistical reliability.

The error range of this paper’s algorithm in six environments is 0.39 m–1.20 m, which shows good environmental adaptability and robustness of perceptual fusion. Although the PF algorithm is slightly better than this paper’s algorithm in terms of error, the gap in most tests is not significant. In terms of the computational resource consumption, it is much higher than other methods. UKF performs slightly better than this paper’s algorithm in some highly nonlinear scenarios, but the lack of multi-sensor weight fusion capability causes its error to increase rapidly in harsh environments, up to 1.38 m. The path integral method, on the other hand, has consistently higher errors (up to 2.43 m) due to the absence of an external observation correction mechanism and expands rapidly with the terrain ups and downs and meteorological deterioration.

In addition, in terms of average time consumption, the overall computational complexity of this paper’s algorithm is kept at O(n) level due to the fact that it only needs to perform one Kalman update and the fusion weight preprocessing module is a matrix-weighting operation. In contrast, UKF needs to propagate 2n + 1 sigma points, and PF needs to maintain hundreds or even thousands of particles, which are 1.46 times and 8.0 times of the algorithm of this paper in terms of time consumption, respectively. Therefore, the algorithm in this paper is more suitable for embedded system deployment with high real-time requirement while maintaining high accuracy.

### 4.3. Computational Efficiency Evaluation

In order to verify the stability of the algorithm under complex perturbation conditions, this paper analyzes both theoretical and experimental aspects. First, the state error is constructed as follows:

(19) $$e(t)=x_{\mathrm{true}}(t)-\hat{x}(t)$$

where $x_{\mathrm{true}}(t)$ is the true state of the system at moment *t*; $\hat{x}(t)$ is the filter’s estimate of the state.

Construct the Lyapunov candidate function as follows:

(20) $$V(t)=e{\left(t\right)}^{T}P^{-1}e(t)$$

where *P* is the state covariance matrix.

Differentiating the state update formulation of the EKF yields that the system is asymptotically stable if there exists *η* > 0 such that $\frac{dV(t)}{dt}\;<\;-\eta ∥e(t){∥}^{2}$ holds.

The calculation under each scenario yields *η* > 0 and the error bound converges, proving that the filtering process does not diverge.

The stability of the algorithm is further verified using Monte Carlo simulation. The error variance of this paper’s algorithm is lower than that of the UKF and the path integral method, while its confidence interval is narrower and close to that of the PE algorithm, indicating that the estimation results are more concentrated and more stable. This result proves that the bionic adaptive mechanism can effectively deal with the environmental perturbations and avoid the dispersion of state estimation. Although the time complexity of the algorithm is higher than that of the path integral algorithm due to the introduction of the dynamic adjustment mechanism, the single-step execution time is only 26% higher than that of the path integral algorithm and much lower than that of the PF algorithm, which is extremely time-consuming. Although the computational efficiency of the algorithm is slightly increased compared with the path integral algorithm, the single-step execution time is still controlled within 1 ms, which fully meets the real-time control requirements of 10 Hz for plant protection machines. This small computational overhead is exchanged for the significant improvement in accuracy in complex environments, which reflects the rationality of the design of the algorithm and its applicability to engineering. In the future, the execution time can be further shortened by hardware acceleration or code optimization to meet the demand of higher frequency control.

## 5. Results

The bionic EKF algorithm proposed in this paper is optimized by sensor fusion to achieve high positioning accuracy in complex agricultural environments. Experimental data show that the average position error in hilly and windy rainy scenarios is reduced by 24% compared to the UKF algorithm and 50% compared to the path integration algorithm, while the single-step execution time consumption is still controlled within 1 ms, which fully meets the real-time control requirements of 10 Hz for plantation machines, with lower error variance and more stable performance. Its design is inspired by the multimodal information integration strategy of migratory birds, which demonstrates the advantages of bionic strategy in dealing with complex scenes. The slight sacrifice in computational efficiency of the algorithm is exchanged for a significant increase in robustness, which provides reliable technical support for the autonomous navigation of agricultural plant protection machines.

In summary, the bionic fusion EKF algorithm proposed in this paper balances positioning accuracy, perceptual adaptivity, and computational efficiency in unstructured and multi-disturbance environments and is suitable for the following complex navigation environments:

1. Forestry monitoring: e.g., fire patrol in mountainous areas, mapping of pest and disease distribution;
2. Detection of disaster areas: such as semi-autonomous search and localization of collapsed areas after earthquakes;
3. Special terrain transportation robots: such as snow, desert, and other environments in the path estimation.

All of the above applications require stable navigation in scenes with large sensor uncertainty and poor visibility, and the bionic EKF provides an effective solution for this.

## Figures and Tables

**Table 1:** Dynamic adjustment of parameters.

Environmental Combinations	Maximum Adjustment Factor
plains + sunny	0.05
plains + heavy rain	0.20
plains + windy and rainy	0.35
hills + sunny	0.12
hills + heavy rain	0.35
hills + windy and rainy	0.50

**Table 2:** Noise amplification factor.

Environmental Combinations	Title 2	Title 3	Title 4
plains + sunny	1.0	1.0	1.0
plains + heavy rain	3.0 + random perturbation	1.0	1.0
plains + windy and rainy	3.0 + random perturbation	4.0 + random perturbation	2.8 + random perturbation
hills + sunny	5.0	1.0	3.2
hills + heavy rain	5.0 × 3.0	1.0	3.2 × 1.0
hills + windy and rainy	5.0 × 3.0	4.0 + random perturbation	3.2 × 2.8

**Table 3:** Comparison of algorithm time consumption.

Arithmetic	Single-Step Time-Consuming (Milliseconds)
Bio-EKF	0.97
UKF	1.42
PF	7.83
Path	0.71

**Table 4:** Comparison of error analysis.

Arithmetic	MSE (m^2^)	Width of 95% Confidence Interval (m)
Bio-EKF	0.39	1.13
UKF	0.80	1.84
PF	0.20	0.98
PATH	3.86	2.35

## Data Availability

Dataset available on request from the authors.
